# Never

**Published:** 1877-01

**Authors:** 


### NEVER

NEVER taste an atom when you are not hungry; it is suicidal.

Never enter an omnibus without having the exact change.

Never stop to talk in a church aisle after service is over.

Never hire servants who go in pairs, as sisters, cousins, or any thing else.

Never blow your nose between your thumb and fingers.

Never deposit the results of a "hawk" or cough on the sidewalk.

Never pick your nose and look at it.

Never open your handkerchief to inspect the product of a "blow."

Never speak of your father as "the old man."

Never reply to the epithet of a drunkard, a fool, or a fellow.

Never speak contemptuously of womankind.

Never abuse one who was once your bosom-friend, however bitter now.

Never smile at the expense of your religion or your Bible

Never stand at the corner of a street.

Never take a second nap.

Never eat a hearty supper.

Never insult poverty.

Never eat between meals.

